# Severe, Resistant Generalized Pustular Psoriasis-Associated Inflammatory Syndrome Successfully Treated With Adalimumab: A Case Report

**DOI:** 10.7759/cureus.98830

**Published:** 2025-12-09

**Authors:** Evangelos Bourousis, Marina Triantafyllia Kotzamani, Despoina N Maritsi

**Affiliations:** 1 Paediatrics and Child Health, Paediatric Hospital, Athens, GRC; 2 Paediatrics, National and Kapodistrian University of Athens, Athens, GRC

**Keywords:** adalimumab (humira), generalized pustular psoriasis (gpp), genetics of psoriasis, pediatric psoriasis, pediatric rheumatology

## Abstract

Generalized pustular psoriasis (GPP) is a rare and severe variant of psoriasis, especially in pediatric patients, characterized by recurrent episodes of fever, malaise, and sterile pustules. We present the case of an eight-year-old girl with genetically confirmed GPP, carrying heterozygous mutations in IL36RN and CARD14, successfully treated with adalimumab in combination with methotrexate. Despite previous therapies, sustained remission was achieved only with this regimen. This case highlights the importance of genetic testing and the potential role of biologic agents in the management of pediatric GPP.

## Introduction

Psoriasis is a chronic, immune-mediated inflammatory disease affecting approximately 2-3% of the global population [1]. Among its clinical variants, generalized pustular psoriasis (GPP) is one of the rarest and most severe, accounting for less than 1% of psoriasis cases [2]. GPP is characterized by recurrent flares of widespread sterile pustules on erythematous skin, often accompanied by systemic inflammation, including high fever, malaise, arthralgia, and laboratory evidence of systemic inflammation [3].

Childhood-onset GPP is particularly uncommon, with most cases presenting within the first year of life [4]. The disease may follow a relapsing course with potentially life-threatening complications such as sepsis, hepatic dysfunction, and electrolyte imbalance if untreated [5]. Early diagnosis is therefore crucial but remains challenging due to clinical overlap with severe infections and other pustular dermatoses.

Recent advances have identified a strong genetic component in GPP. Mutations in the IL36RN gene, first described in 2011 [6], lead to defective interleukin-36 (IL-36) receptor antagonism and uncontrolled pro-inflammatory signaling. Additional mutations such as CARD14, BTN3A3, AP1S3, and SERPINA1 have also been implicated [6]. These discoveries highlight GPP as a distinct autoinflammatory disease entity, rather than merely a variant of plaque psoriasis.

Traditional therapies, including systemic corticosteroids, cyclosporine, methotrexate, and retinoids, have shown variable efficacy and are often associated with significant adverse effects in children [7]. The introduction of biologic agents, particularly tumor necrosis factor-alpha (TNF-α) inhibitors and, more recently, IL-36 receptor antagonists, has provided promising new options, although high-quality pediatric data remain limited [8,9].

Here, we present the case of an eight-year-old girl with genetically confirmed GPP due to IL36RN and CARD14 mutations, who demonstrated dramatic and sustained clinical remission following treatment with adalimumab. This case emphasizes the diagnostic value of genetic testing and the potential role of biologics in pediatric GPP.

## Case presentation

An eight-year-old Afghan girl, born from a non-consanguineous marriage, presented with recurrent episodes of high-grade fever, malaise, and widespread erythematous, scaly pustular eruptions. Symptoms had first appeared at six months of age and persisted intermittently, despite multiple systemic treatments. Prior to referral, the patient had received topical corticosteroids, oral retinoids, cyclosporine, and methotrexate, with only partial and short-lived responses.

**Figure 1:**
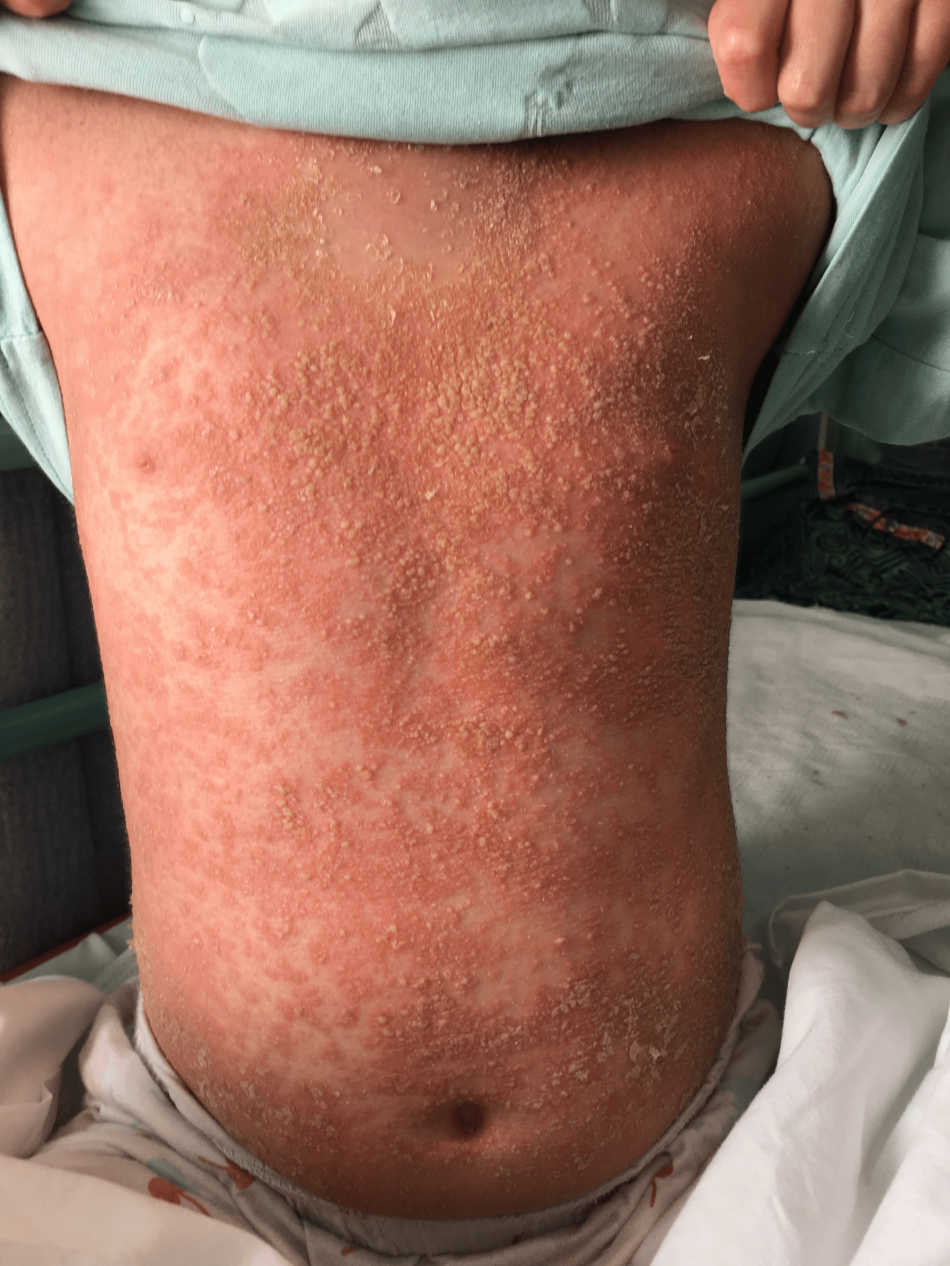
Generalized erythematous and scaly pustular lesions involving the trunk and extremities before treatment with adalimumab.

Upon admission to our institution, she was febrile (39.5°C), alert but fatigued, and displayed a generalized pustular and scaly rash involving the face, trunk, and extremities. Clinical appearance at presentation is shown in Figure 1. Laboratory evaluation revealed leukocytosis, anemia, thrombocytosis, and markedly elevated inflammatory markers, including C-reactive protein (CRP) at 98 mg/L and erythrocyte sedimentation rate (ESR) at 57 mm/h.

Given the atypical presentation and chronic course, an extensive work-up was performed to exclude infectious or immunologic mimickers. Blood cultures, skin lesion cultures, urine cultures, Mantoux test, and T-SPOT.TB were negative. Ophthalmologic and cardiac evaluations were unremarkable. Skin biopsy confirmed the diagnosis of pustular psoriasis. Whole-exome sequencing revealed heterozygous mutations in the IL36RN (c.368C>T) and CARD14 genes.

Management was initiated with prednisolone and anakinra (IL-1 receptor antagonist), resulting in modest clinical improvement. However, the patient experienced a flare during hospitalization, with reappearance of pustular lesions and recrudescence of fever and CRP elevation. As a result, anakinra was discontinued, and treatment was escalated to adalimumab (0.8 mg/kg subcutaneously every two weeks) in combination with methotrexate (10 mg weekly). Prednisolone was tapered and discontinued.

**Figure 2:**
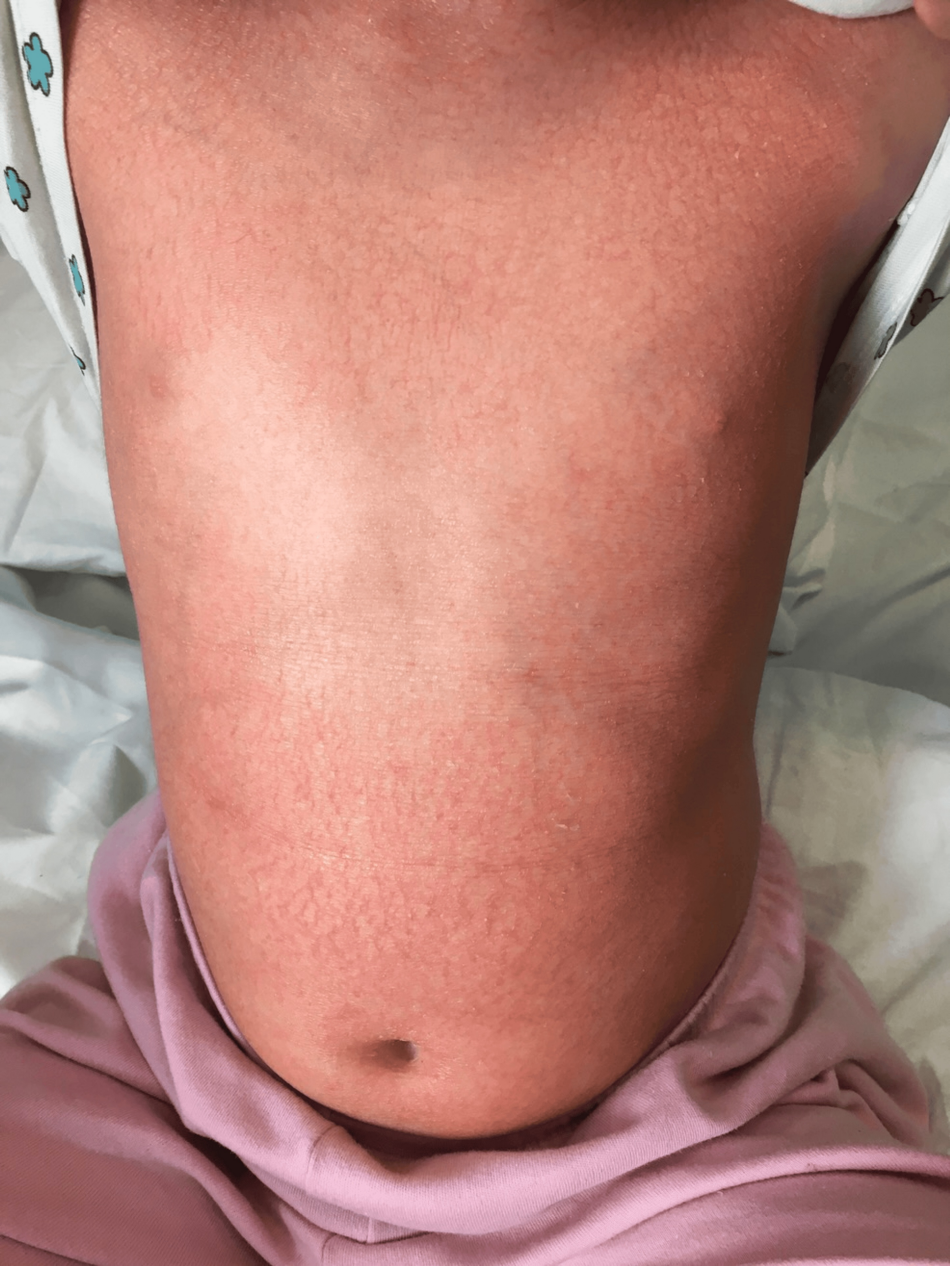
Marked clinical improvement two weeks after initiation of adalimumab.

Within days of initiating adalimumab, the patient became afebrile and demonstrated marked cutaneous improvement. By the end of the second week, the pustular rash had almost fully resolved, with only minimal residual scaling. Clinical response at two weeks is shown in Figure 2. The patient was discharged on day 35 of hospitalization and has since remained in sustained remission for 12 months without clinical flares. A summary of laboratory parameters at admission, at two weeks post-treatment, and at discharge is provided in Table 1.

**Table 1:** Laboratory parameters during the clinical course of pediatric generalized pustular psoriasis (GPP). WBC: white blood cell; Hb: hemoglobin; PLT: platelet count; CRP: C-reactive protein; ESR: erythrocyte sedimentation rate

Timepoint	WBC (/μL)	Hb (g/dL)	PLT (/μL)	CRP (mg/L)	ESR (mm/h)
Admission	13,600	9.8	258,000	98	57
After two weeks	10,700	10	402,000	50	45
Discharge	9,400	10.1	601,000	1	20

## Discussion

GPP is a rare but potentially life-threatening condition. To date, multiple genetic mutations have been linked to GPP. In our patient, the presence of IL36RN and CARD14 mutations suggests a genetic predisposition contributing to disease severity. Laboratory results demonstrated significant improvement within two weeks of initiating adalimumab and methotrexate, with normalization of inflammatory markers and sustained remission for 12 months. The pathogenesis of GPP has been increasingly linked to genetic mutations such as IL36RN, first described in 2011 [4]. Additional variants, including CARD14, BTN3A3, and AP1S3, have also been implicated [5,6]. These mutations are believed to enhance IL-36-mediated inflammatory pathways, which may contribute to pustule formation in genetically susceptible individuals. Traditional therapies such as cyclosporine, methotrexate, and acitretin have limited effectiveness in pediatric GPP [3,5]. Biologic agents, including TNF-α inhibitors like adalimumab, have shown promising results [7,8]. Recently, IL-36 receptor antagonists such as spesolimab have been approved for use in adolescents [9], and imsidolimab is under phase III clinical trials [10]. Our case underlines the role of genetic testing, not only for diagnosis, but also for therapeutic decision-making. Genetic confirmation may assist in selecting targeted therapies, particularly in cases resistant to conventional immunosuppressants. While targeted immune therapies show clear promise, randomized controlled trials in pediatric populations remain scarce. This case report represents a single-patient observation and thus cannot establish causality. Furthermore, as methotrexate was co-administered with adalimumab, its contributory role to disease remission cannot be excluded.

## Conclusions

We present a case of pediatric GPP with heterozygous IL36RN and CARD14 mutations, demonstrating a favorable and sustained response to the combination of adalimumab and methotrexate. This report highlights the potential role of early genetic evaluation in guiding treatment decisions, particularly in refractory cases. Further prospective studies are warranted to evaluate the safety, efficacy, and optimal sequencing of biologic therapies in genetically defined subgroups of pediatric GPP.

## References

[1] Zheng M, Jullien D, Eyerich K (2022). The prevalence and disease characteristics of generalized pustular psoriasis. Am J Clin Dermatol.

[2] Chen Y, Xiang X, Wang Z, Miao C, Xu Z (2023). The update of treatment strategies in pediatrics with generalized pustular psoriasis in China. Pediatr Investig.

[3] de Oliveira ST, Maragno L, Arnone M, Fonseca Takahashi MD, Romiti R (2010). Generalized pustular psoriasis in childhood. Pediatr Dermatol.

[4] Onoufriadis A, Simpson MA, Pink AE (2011). Mutations in IL36RN/IL1F5 are associated with the severe episodic inflammatory skin disease known as generalized pustular psoriasis. Am J Hum Genet.

[5] Rivera-Díaz R, Daudén E, Carrascosa JM, Cueva P, Puig L (2023). Generalized pustular psoriasis: a review on clinical characteristics, diagnosis, and treatment. Dermatol Ther (Heidelb).

[6] Yang SF, Lin MH, Chou PC, Hu SK, Shih SY, Yu HS, Yu S (2023). Genetics of generalized pustular psoriasis: current understanding and implications for future therapeutics. Genes (Basel).

[7] Menter A, Cordoro KM, Davis DM (2020). Joint American Academy of Dermatology-National Psoriasis Foundation guidelines of care for the management and treatment of psoriasis in pediatric patients. J Am Acad Dermatol.

[8] Megna M, Camela E, Ruggiero A, Battista T, Martora F, Cacciapuoti S, Potestio L (2023). Use of biological therapies for the management of pustular psoriasis: a new era?. Clin Cosmet Investig Dermatol.

[9] Bernardo D, Thaçi D, Torres T (2024). Spesolimab for the treatment of generalized pustular psoriasis. Drugs.

[10] Warren RB, Reich A, Kaszuba A (2023). Imsidolimab, an anti-interleukin-36 receptor monoclonal antibody, for the treatment of generalized pustular psoriasis: results from the phase II GALLOP trial. Br J Dermatol.

